# αA-Crystallin Mediated Neuroprotection in the Retinal Neurons Is Independent of Protein Kinase B

**DOI:** 10.3389/fnins.2022.912757

**Published:** 2022-05-20

**Authors:** Madhu Nath, Patrice Elie Fort

**Affiliations:** ^1^Department of Ophthalmology and Visual Sciences, University of Michigan, Ann Arbor, MI, United States; ^2^Department of Molecular and Integrative Physiology, University of Michigan, Ann Arbor, MI, United States

**Keywords:** αA-crystallin, protein kinase B, retinal neurons, apoptosis, neuroprotection

## Abstract

Phosphatidylinositol 3-kinase (PI3K)/Akt signal pathway mediates pro-survival function in neurons. In the retina, PI3K/AKT/mTOR signaling pathway is related to the early pathogenesis of diabetic retinopathy. Signaling molecules in the membrane-initiated signaling pathway exhibiting neuroprotective function interacts with the PI3K/Akt pathway as an important survival pathway. Molecular chaperone α-crystallins are known to potentially interact and/or regulate various pro-survival and pro-apoptotic proteins to regulate cell survival. Among these demonstrated mechanisms, they are well-reported to regulate and inhibit apoptosis by interacting and sequestrating the proapoptotic proteins such as Bax and Bcl-Xs. We studied the importance of metabolic stress-induced enhanced Akt signaling and αA-crystallin interdependence for exhibiting neuroprotection in metabolically challenged retinal neurons. For the first time, this study has revealed that αA-crystallin and activated Akt are significantly neuroprotective in the stressed retinal neurons, independent of each other. Furthermore, the study also highlighted that significant inhibition of the PI3K-Akt pathway does not alter the neuroprotective ability of αA-crystallin in stressed retinal neurons. Interestingly, our study also demonstrated that in the absence of Akt activation, αA-crystallin inhibits the translocation of Bax in the mitochondria during metabolic stress, and this function is regulated by the phosphorylation of αA-crystallin on residue 148.

## Introduction

Protein kinase B (Akt) is an essential signaling protein that can be activated by various growth factors such as platelet-derived growth factor, epidermal growth factor, basic fibroblast growth factor, and insulin-like growth factor as well as insulin (Fayard et al., 2005; Sale and Sale, 2008). Akt phosphorylation is one of the essential signaling events responsible for various physiological functions, including metabolism, survival/apoptosis, differentiation, and proliferation (Brazil et al., 2004). Various studies have reported that many signaling molecules in the membrane-initiated signaling pathway exhibiting neuroprotective function interact with the PI3K/Akt pathway as an important survival pathway (Mannella and Brinton, 2006). Akt activation can also activate downstream signaling pathways involved in inflammation, such as the induction of phosphorylation and activation of IκB and the release of NF-κB (Ihekwaba et al., 2004). In addition, studies have reported that Akt activation exerts a neuroprotective effect in neuronal cells against ischemic brain damage (Ohba et al., 2004) and oxidative damages in the retina (Yu et al., 2006), besides enhancing the retina insulin receptor response through intraocular insulin growth factor-2 administration in diabetic rats (Zolov et al., 2021).

Pro-survival mechanisms are particularly important for neurons, as they often get exposed to environmental stressors. Retinal neurons consistently deal with external stimuli, which eventually cause oxidative and nutrient stresses, resulting in retinal pathology. Importantly, these noxious environmental stressors can originate from the retina, the adjacent vitreous body, the extracellular matrix, or the blood vessels and capillaries (Skeie and Mahajan, 2013; Thanos et al., 2014). Several groups, including ours, have reported the upregulation of the small heat shock proteins α-crystallins in the vitreous-retina complex of mice (Skeie and Mahajan, 2013) and diabetic donors (Ruebsam et al., 2018). Moreover, the upregulation of α-crystallins is believed to be a part of an adaptive response for promoting neuroprotection (Vazquez-Chona et al., 2004; Ying et al., 2008; Ruebsam et al., 2018). While believed to be associated with the chaperone function, α-crystallins specific neuroprotection mechanisms remain largely unclear.

α-Crystallins are known to potentially interact and/or regulate various pro-survival and pro-apoptotic proteins to regulate cell survival. Among these demonstrated mechanisms are the well-reported regulation and inhibition of apoptosis by interacting with and sequestrating proapoptotic proteins such as Bax and Bcl-Xs (Mao et al., 2004). In lens epithelial cells, αB-crystallin has also been reported to promote survival by activating RAF/MEK/ERK pathway (Liu et al., 2004). Despite demonstrating a strong neuroprotective role in the retina, αA-crystallin interaction with pro-survival signaling pathways is unclear. Since it has been previously shown to potentially regulate Akt signaling in lens epithelial cells, the current study was carried out to delineate the possible interaction of αA-crystallin with pro-survival Akt signaling molecule for its neuroprotective function in retinal neurons under metabolic stress.

## Materials and Methods

### Cell Culture

Rat retinal neuronal cells (R28) were obtained from Applied Biological Materials Inc. (Richmond, BC, Canada). Cells were maintained in DMEM, 5 mM Glucose (DMEM-NG) supplemented with 10% FBS (Flow Laboratories) at 37°C, 5% CO_2_ unless stated otherwise. For experiments, R28 cells were differentiated into neurons in DMEM with 8-(4-Chlorophenylthio)adenosine 3′,5′-cyclic monophosphate (8-CPT-cAMP, Catalog # C3912, Millipore Sigma, St. Louis, MO, United States) at a final concentration of 2.5 mM on laminin-coated plates as described earlier (Ruebsam et al., 2018).

### Transfection and Experimental Protocol

Cells were transfected using the Neon Transfection System (Invitrogen, Waltham, MA, United States) following the manufacturer’s instructions. Briefly, cells were trypsinized and washed in PBS before resuspending in suspension buffer and electroporated with targeted plasmids. Cells were then plated in six-well plates for protein expression studies. Post-transfection, cells were plated in DMEM with 5 or 25 mM glucose for 24 h. The cells were then incubated in either serum-free DMEM, 25 mM glucose, or 25 mM glucose with 100 ng/ml TNFα (R&D Systems, Catalog # 210-TA) for 4 h before analysis, whereas 5 mM DMEM served as the experimental control.

### Cell Death Analysis

To investigate the effect of conditioned media on R28 cell viability, cell death rates were assessed using DNA Fragmentation ELISA (Roche Diagnostics, Indianapolis, IN, United States) according to the manufacturer’s instructions and as previously described (Ruebsam et al., 2018). Transfected R28 cells were seeded in a 96 well plate at a density of 1 × 10^5^ cells per well were incubated with or without stressors and either Akt Inhibitor V or Akt Inhibitor XII (30 μM; Millipore Sigma, Darmstadt, Germany) for 4 h. Following stress, cells were lysed in 100 μl of lysis buffer. Next, 20 μl of the supernatant and the positive and negative controls were transferred into the ELISA plate and the immunoreagent complex. Following incubation and washes, the colorimetric solution was added and incubated until the colorimetric reaction developed. After adding the stop solution, the colorimetric signal was measured with a fluorescence plate reader in a FLUOstar OMEGA plate reader (BMG LABTECH, Ortenberg, Germany) with excitation at 405 and 490 nm.

### Caspase-3/7 Activity Assay

Caspase-3/7 activity was measured using the Apo-ONE Assay (Promega, Madison, WI, United States) described previously (Abcouwer et al., 2010). Briefly, R28 retinal neuron cells were seeded in a 96 well plate at a density of 1 × 10^5^ cells per well were incubated with 100 μl of medium without serum for 4 h. Following incubation, caspase-3/7 activity was measured in the supernatants in a 96-well plate format, according to the manufacturer’s protocol at 37°C.

### Subcellular Fractionation

To assess Bax translocation in mitochondria, R28 retinal neuron cells were subjected for subcellular fractionation. Briefly, the cell pellets were resuspended in 100 μL of cytosolic buffer containing 1X PBS, 300 mM sucrose, 5 mM PMSF, and protease inhibitor cocktail and sonicated. After incubating the mixture at ice for 30 min, the lysates were centrifuged for 60 min at 10,000 × *g* at 4°C, and the supernatant was collected containing cytosolic fraction. The pellet was then resuspended in mitochondrial buffer containing 1X PBS, 1% Triton X-100 150 nM NaCl, and protease inhibitor cocktail and sonicated. The resuspension was centrifuged at 10,000 × *g* for 30 min at 4°C, and the supernatant was collected containing mitochondrial fraction.

### Immunoblot

Cells were homogenized by sonication in the previously described RIPA buffer (Ruebsam et al., 2018). Protein concentrations were measured with the Pierce BCA reagent, and all samples were adjusted for equal protein concentration. Whole lysates and subcellular fractions were immunoblotted using NuPage gels 4–12% and MES buffer following the manufacturer’s instructions (Thermo Fisher Scientific, Waltham, MA, United States). Gels were run in MES buffer (Thermo Fisher Scientific, Waltham, MA, United States) per the manufacturer’s instructions. Western blot transfer was carried out on Nitrocellulose membranes using the Mini Trans-Blot cell (Catalog # 1703930, Bio-Rad, Hercules, CA, United States) at 160 V for 1 h at 4°C. Cell lysates were screened for Akt (9272S, Cell Signaling Technology, Danvers, MA, United States), Bax (D2E11, Cell Signaling Technology, United States), HA Tag (C29F4, Cell signaling technology, United States), phosphorylated-FOXO3a (Cat no #9466, Cell Signaling Technology, United States), phosphorylated-S6 (D68F8, Cell Signaling Technology, United States), phosphorylated-4EBP1 (236B4, Cell Signaling Technology, United States), Total-4EBP1 (53H11, Cell Signaling Technology, United States), GAPDH (D16H11, Cell Signaling Technology, United States), COX-IV (Cat no #4844, Cell Signaling Technology, United States), αA-crystallin (sc-28306, Santa Cruz Biotechnology, Dallas, TX, United States) expression and β-actin (MAB-1501, Millipore, Burlington, MA, United States) as a loading control.

### Statistics

The mean ± SEM and statistically significant differences are reported. Analyses were performed using non-repeated-measures ANOVA, followed by the Student–Newman–Keuls test for multiple comparisons. A *p*-value less than 0.05 was considered significant.

## Results

### αA-Crystallin Protects Retinal Neurons From Metabolic Stress by a Mechanism Independent of the Pro-survival Akt Pathway

**FIGURE 1:**
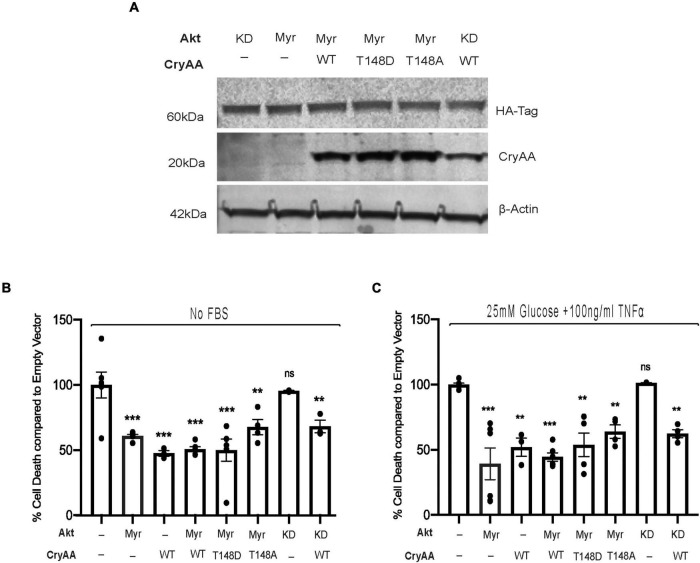
αA-Crystallin protects retinal neurons in stress conditions, independent of the pro-survival Akt pathway. Rat retinal neuronal cells (R28) cells were transfected with either empty vector (EV), wild type αA-crystallin (WT), phosphomimetic form of αA-crystallin (T148D), or non-phosphorylatable form of αA-crystallin (T148A) with/without myristoylated (Myr) or kinase-dead (KD) form of protein kinase B (Akt). Post transfection, cells were either kept in serum starvation (No FBS) or exposed to diabetic-like stress (25 mM glucose + 10 ng/ml TNFα). The expression of αA-crystallin and Akt in cell lysates was assessed for stressed R28 cells **(A)** using immunoblotting. DNA fragmentation ELISA was performed as the endpoint on Rat retinal neuronal cells (R28), incubated with the No FBS **(B)** or diabetic-like stress **(C)** for 4 h. Each endpoint was measured on a minimum of three technical replicates in two independent experiments (*n* = 3/each condition). Statistical analysis was performed by one-way ANOVA followed by the Student–Newman–Keuls test. **p ≤ 0.01, ***p ≤ 0.001. CryAA, αA-crystallin; Akt, protein kinase B; TNFα, tumor necrosis factor-alpha; HA-Tag, human influenza hemagglutinin tag; Myr, myristoylated form of protein kinase B; KD, a kinase-dead form of protein kinase B; WT, wild type αA-crystallin; T148D, phosphomimetic form of αA-crystallin; T148A, non-phosphorylatable form of αA-crystallin.

We have previously demonstrated that αA-crystallin is strongly neuroprotective for retinal neurons exposed to metabolic stress and that threonine 148 phosphorylation essentially controls the protective role of αA-crystallin (Ruebsam et al., 2018). We indeed reported that while the phosphomimetic form of αA-crystallin shows improved neuroprotective function, the non-phosphorylatable form showed an almost complete lack of protection, consistent with a key regulatory function of this phosphorylation (Ruebsam et al., 2018). Since αA-crystallin was previously shown to promote epithelial cell survival through modulation of the pro-survival Akt pathway, we aimed to test the existence of a similar relationship in retinal neurons. For this analysis, we have used differentiated retinal neurons from rat R28 retinal neuron cells overexpressing either myristoylated (Myr) or kinase-dead (KD) form of Akt with or without wild type (WT) phosphomimetic (T148D) or non-phosphorylatable form (T148A) of αA-crystallin (Figure 1A). Our data demonstrated that retinal neurons overexpressing the myristoylated (Myr-Akt) form of Akt or the wild type (WT) form of αA-crystallin alone had ∼50% reduction in cell death induced by serum starvation (Figure 1B) and “diabetic-like” stress (Figure 1C). No additional protection was observed in retinal neurons co-overexpressing Myr-Akt with either WT or the phosphomimetic form of αA-crystallin in either metabolic stress (Figures 1B,C). As expected, our data showed that retinal neurons overexpressing the non-phosphorylatable αA-crystallin mutant (not shown) or the kinase-dead form of Akt had cell death levels comparable to that of the empty vector-transfected cells. Suggestive of independent mechanisms, overexpression of the KD form of Akt did not impact the protective effect of αA-crystallin overexpression. In contrast, overexpression of the non-phosphorylatable αA-crystallin mutant did not impact the protective effect of the myristoylated Akt in either the serum starvation or “diabetic-like” stress conditions (Figures 1B,C).

### Pan-Akt Inhibition Does Not Impact the Protective Effect of αA-Crystallin Overexpression

**FIGURE 2:**
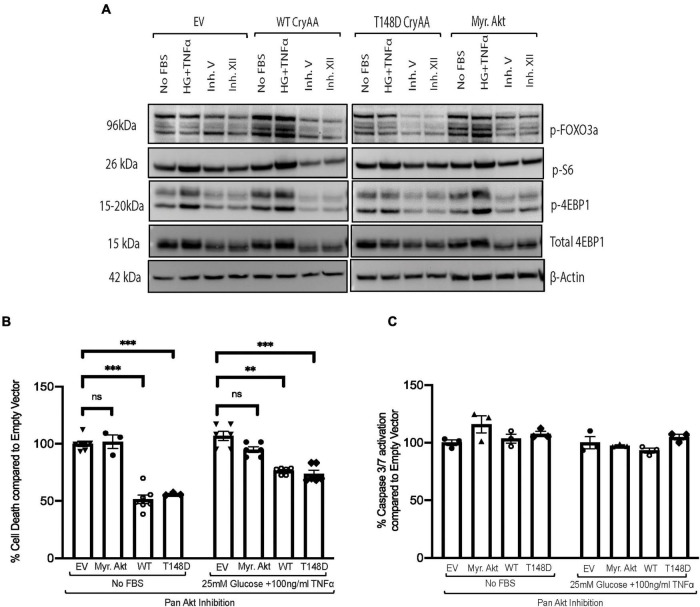
Pan-Akt inhibition does not impact the protective effect of αA-crystallin on retinal neurons. Rat retinal neuronal cells (R28) cells were transfected with either empty vector (EV), wild type αA-crystallin (WT), phosphomimetic form of αA-crystallin (T148D), or myristoylated (Myr) form of protein kinase B (Myr-Akt). For pharmacological inhibition of Pan-Akt expression, post transfection, cells were either kept in serum starvation (No FBS) or exposed to diabetic-like stress (25 mM glucose + 10 ng/ml TNFα) with the pan-Akt inhibitor XII (30 μM conc.) for 4 h. The expression of downstream targets of the Akt pro-survival pathway in cell lysates was assessed for stressed R28 cells **(A)** using immunoblotting. DNA fragmentation ELISA **(B)** and caspase 3/7 activation **(C)** assay were performed as the endpoint on Rat retinal neuronal cells (R28), incubated with the No FBS or diabetic-like stress **(B,C)** for 4 h, respectively. Each endpoint was measured on a minimum of three technical replicates in two independent experiments (*n* = 3/each condition). Statistical analysis was performed by one-way ANOVA followed by the Student–Newman–Keuls test. **p ≤ 0.01, ***p ≤ 0.001. CryAA, αA-crystallin; Akt, protein kinase B; TNFα, tumor necrosis factor-alpha; HA-Tag, human influenza hemagglutinin tag; Myr, myristoylated form of protein kinase B; KD, a kinase-dead form of protein kinase B; WT, wild type αA-crystallin; T148D, phosphomimetic form of αA-crystallin; T148A, non-phosphorylatable form of αA-crystallin; p-FOXO3a, phosphorylated forkhead box O-3; p-S6, phosphorylated ribosomal protein S6; p-4EBP1, phosphorylated factor 4E-binding protein 1.

We then used a chemical inhibitor-based approach as a secondary method to assess the involvement of Akt in the regulation of αA-crystallin neuroprotective effect and its regulation by T148 phosphorylation. Because R28 retinal neurons endogenously express multiple Akt isoforms, differentiated R28 retinal neurons overexpressing the WT or phosphomimetic form of αA-crystallin were exposed to serum starvation or “diabetic-like” stress in the presence of a pan-Akt inhibitor (XII) that we previously characterized (Gardner et al., 2015). As we previously showed, this pan-Akt specific inhibitor dramatically reduced the phosphorylation of proteins downstream of Akt, including FOXO3a, S6, and 4E-BP1 (Figure 2A), confirming the significant inhibition of Akt signaling. Furthermore, specific Akt signaling inhibition by this treatment was confirmed by its effectiveness in suppressing the protective impact of Myr-Akt on cell death induced by either of these metabolic stress conditions (Figure 2B). Consistent with parallel effects of these protective pathways, our data further revealed that inhibition of Akt had no impact on the protective effect of overexpression of either the WT or phosphomimetic form of αA-crystallin (Figure 2B). Of note, while not impacting cell death, inhibition of endogenous Akt has led to increasing caspase 3/7 activity in all the groups and was comparable to the empty vector (Figure 2C), suggesting that the protective effect of αA-crystallin is at least partially caspase-independent.

### αA-Crystallin Phosphorylation at Residue T148 Inhibits Stress-Induced Bax Translocation to Mitochondria Independent of Pro-survival Akt Pathway

**FIGURE 3:**
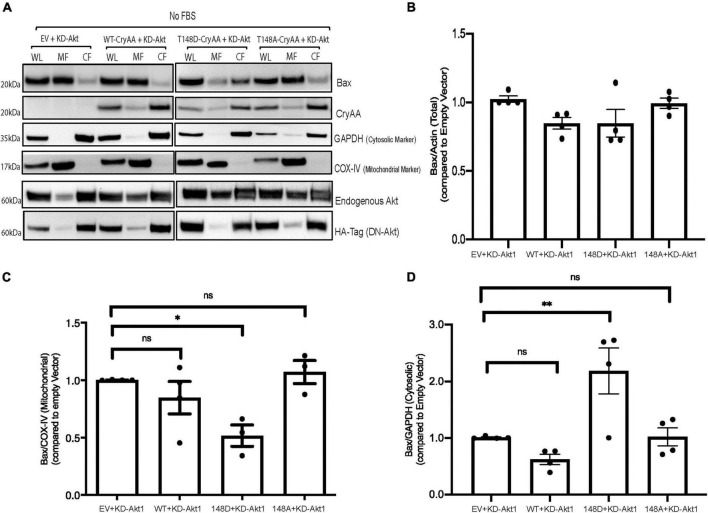
αA-Crystallin phosphorylation at residue 148D is important for inhibiting stress-induced Bax translocation to mitochondria and does not depend on Akt to mediate this effect. Rat retinal neuronal cells (R28) cells were co-transfected with either empty vector (EV), wild type αA-crystallin crystallin (WT), phosphomimetic form of αA-crystallin (T148D), or non-phosphorylatable form of αA-crystallin (T148A) with kinase-dead (KD) form of Akt. Post transfection, cells were exposed to serum starvation (No FBS) for 4 h. Bax, CryAA, and kinase-dead Akt expression in cytosolic, mitochondrial, and whole lysates were assessed in stressed R28 cells **(A)** using immunoblotting. In addition, the expression of Bax in total lysates **(B)**, the mitochondrial fraction **(C)**, and cytosolic fractions **(D)** were analyzed using ImageJ software. Each endpoint was measured on a minimum of three technical replicates in two independent experiments (*n* = 3/each condition). Statistical analysis was performed by one-way ANOVA followed by the Student–Newman–Keuls test. *p ≤ 0.05, **p ≤ 0.01. CryAA, αA-crystallin; Akt, protein kinase B; GAPDH, glyceraldehyde 3-phosphate dehydrogenase; COX-IV, cytochrome c oxidase complex IV; HA-Tag, human influenza hemagglutinin tag; KD, a kinase-dead form of protein kinase B; WT, wild type αA-crystallin; T148D, phosphomimetic form of αA-crystallin; T148A, non-phosphorylatable form of αA-crystallin.

We previously reported that αA-crystallin prevents neuronal cells death through regulation of Bax translocation to the mitochondria. Thus, we next assessed how manipulation of Akt signaling might impact the regulation of the cellular localization of Bax by αA-crystallin. Differentiated R28 retinal neurons overexpressing dominant negative Akt with either WT, phosphomimetic, or non-phosphorylatable form of αA-crystallin exposed to metabolic stress was analyzed for Bax expression and subcellular localization (Figure 3A). We first confirmed that the presence of KD Akt with either form of αA-crystallin in metabolically stressed retinal neurons does not have any impact on the total Bax expression (Figure 3B). Consistent with a key role of T148 phosphorylation independent of Akt, the metabolic stress-induced translocation of Bax to the mitochondria was almost completely suppressed in retinal neurons overexpressing the phosphomimetic form of αA-crystallin, even when co-expressing the KD form of Akt (Figures 3C,D). Altogether this study demonstrates that αA-crystallin protects retinal neurons from metabolic stress-induced cell death by sequestrating Bax in a T148 phosphorylation-dependent manner independent of Akt signaling.

## Discussion

The current work studied the importance of metabolic stress-induced enhanced Akt signaling and αA-crystallin interdependence for exhibiting neuroprotection in metabolically challenged retinal neurons. For the first time, this study has revealed that αA-crystallin and activated Akt are significantly neuroprotective in the stressed retinal neurons, independent of each other. Furthermore, the study also highlighted that significant inhibition of the PI3K-Akt pathway does not alter the neuroprotective ability of αA-crystallin in stressed retinal neurons. Interestingly, our study also demonstrated that in the absence of Akt activation, αA-crystallin inhibits the translocation of Bax in the mitochondria during metabolic stress, and this function is regulated by the phosphorylation of αA-crystallin on residue 148. Overall, our study suggests that αA-crystallin and its phosphorylation on residue 148 plays an important role in regulating apoptosis in stressed retinal neurons and can exert this function independent of Akt signaling.

Phosphatidylinositol 3-kinase (PI3K)/Akt signal pathway is well known for mediating pro-survival function in neurons. In the retina, PI3K/AKT/mTOR signaling pathway is related to the early pathogenesis of diabetic retinopathy (Reiter et al., 2006; Fort et al., 2014; Zhang et al., 2019). This pathway is reported to play a major role in protecting against oxidative stress-induced apoptosis (Faghiri and Bazan, 2010) and high glucose-induced inflammatory injury in retinal pigment epithelial cells (Ran et al., 2019). Studies have also reported its active participation in oxidative stress-induced apoptosis in retinal neurons (Yu et al., 2006) and a cytoprotective role in response to the local redox environment of human RPE cells (Kim et al., 2010).

Previous reports, including ours, have demonstrated that the small heat shock chaperone protein αA-crystallin is considerably neuroprotective to the metabolically stressed neurons (Thanos et al., 2014; Ruebsam et al., 2018; Zhu and Reiser, 2018). While studying αA-crystallin possible interaction with the Akt pathway in demonstrating its neuroprotective ability, our current study revealed that it is neuroprotective to metabolically stress retinal neurons, and this function is independent of Akt activation. α-Crystallins were recently proposed to prevent the oxidative stress-related injury to retinal ganglion cells *via* regulation of the Akt/BAD pathway (Hua Wang et al., 2020), and αB-crystallin was reported to be effectively promoting astrocytes viability through phosphatidylinositol 3-kinase (PI3K)/protein kinase B (Akt) signaling pathways under serum-deprivation (Zhu et al., 2015). Our study further demonstrated that pharmacological inhibition of the PI3K-Akt pathway did not alter the neuroprotective ability of αA-crystallin in the stressed retinal neurons. Supportive of the key role of Akt activation, studies have reported that inhibition of Akt using the inhibitor Akt VIII causes a significant increase in retinal-derived cell death induced by H_2_O_2_ (Wang et al., 2015).

Several groups, including ours, have reported that α-crystallins reduce stress-induced apoptosis in part by interfering with the mitochondrial translocation of the pro-apoptotic protein Bax (Mao et al., 2004; Dou et al., 2012; Hamann et al., 2013). Furthermore, decreased α-crystallin expression during metabolic disease progression is also correlated with enhanced Bax pro-death activity (Hamann et al., 2013). Our group has also reported the strong association of diabetes-associated reduced chaperone function of α-crystallins with the increased disruption of its interactions with Bax, a function also shown to be critical for the neuroprotective effect of α-crystallins in retinal neurons in culture (Losiewicz and Fort, 2011). While αB-crystallin protects retinal pigment epithelial cells from ER stress-induced apoptosis by attenuating the increase in Bax (Dou et al., 2012), the C-terminal extension domain of αA-crystallin was sufficient to protect against Bax-induced apoptosis in cone-derived 661W cells (Hamann et al., 2013). Our current study showed that alteration of the Akt survival pathway by overexpression of the KD mutant did not affect the inhibition of the translocation of Bax to the mitochondria by αA-crystallin and that this function was strongly regulated by the phosphorylation of αA-crystallin on residue 148. It is well documented that crystallins undergo numerous post-translation modifications (PTMs), affecting their chaperone activity (Blakytny et al., 1997; Kamei et al., 1997; Ciano et al., 2016). Our group had previously reported that phosphorylation on the serine/threonine 148 residue is essential to retaining the protective role of αA-crystallin under metabolic stress and diabetic conditions (Ruebsam et al., 2018). The current study demonstrated that αA-crystallin protects retinal neurons during metabolic stress by inhibiting the translocation of Bax to the mitochondria and does so completely independently of the Akt signaling pro-survival pathway and that this function is regulated by the phosphorylation of αA-crystallin on residue 148. This observation further confirms the key regulatory role of phosphorylation on the residue 148 of αA-crystallin for its protective function.

In conclusion, this study underlines the neuroprotective role of αA-crystallin and its modulation by phosphorylation on T148 in suppressing pro-apoptotic Bax independent of the PI3K-Akt pathway. Additionally, these findings indicate a potential implication of αA-crystallin modulation for the plausible treatment of neurodegeneration induced by diabetes.

## Data Availability Statement

The original contributions presented in the study are included in the article/supplementary material, further inquiries can be directed to the corresponding author/s.

## Author Contributions

MN performed the experiments and prepared the figures. MN and PF analyzed the data, conceptualized and designed the research, interpreted the results of the experiments, and drafted, finalized, and approved the manuscript. Both authors contributed to the article and approved the submitted version.

## Conflict of Interest

The authors declare that the research was conducted in the absence of any commercial or financial relationships that could be construed as a potential conflict of interest.

## Publisher’s Note

All claims expressed in this article are solely those of the authors and do not necessarily represent those of their affiliated organizations, or those of the publisher, the editors and the reviewers. Any product that may be evaluated in this article, or claim that may be made by its manufacturer, is not guaranteed or endorsed by the publisher.
